# Light up the embryos: knock-in reporter generation for mouse developmental biology

**DOI:** 10.1590/1984-3143-AR2020-0055

**Published:** 2020-08-05

**Authors:** Bin Gu

**Affiliations:** 1 Program in Developmental and Stem Cell Biology, Hospital for Sick Children, Toronto, Canada; 2 Department of Obstetrics, Gynecology and Reproductive Biology, College of Human Medicine, Michigan State University, Michigan, USA; 3 Institute for Quantitative Health Science and Engineering, Michigan State University, Michigan, USA

**Keywords:** CRISPR-Cas9, genome editing, knock-in reporter, HDR, embryo

## Abstract

Developmental biology seeks to understand the sophisticated regulated process through which a single cell – a fertilized egg – generates a highly organized organism. The most effective way to reveal the nature of these processes is to follow single cells and cell lineages in real-time. Recent advances in imaging equipment, fluorescent tags and computational tools have made long term multi-color imaging of cells and embryos possible. However, there is still one major challenging for achieving live imaging of mammalian embryos- the generation of embryos carrying reporters that recapitulate the endogenous expression pattern of marker genes. Recent developments of genome editing technology played important roles in enabling efficient generation of reporter mouse models. This mini review discusses recent developments of technologies for efficiently generate knock-in reporter mice and the application of these models in live imaging development. With these developments, we are starting to realize the long-sought promises of realtime analysis of mammalian development.

## Main text

‘Seeing is believing.’ There is nothing more convincing in developmental biology than a movie that reveal the progress of embryonic development in realtime, such as this one showed here (movie 1). This movie captures the process when a mouse preimplantation embryo develops from a 16-cell embryo to a blastocyst with a frame captured every 20 minutes. Two cell lineages were marked by distinctive fluorescent reporters of lineage marker genes, Cdx2-eGFP (in green) for trophectoderm(TE) and Halo-Sox2 (in red) for inner cell mass(ICM). As you can see, the movie captured many critical processes including, the morphogenetic transition from a solid spherical morula to a blastocyst that contains a central  cavity- blastocoel, and the sequential emergence of *Cdx2* positive TE and *Sox2* positive ICM cells. You can even tell that the *Sox2* positive cells emerge roughly 9 hours after the *Cdx2* positive cells from the time stamps. With this level of spatio-temporal resolution, developmental biology is entering an exciting time. A few technological advancements are required for developmental biology to enter the live imaging time: First, fluorescent tag proteins need to be developed for visualizing a biological entity such as protein, RNA or DNA in living embryos. There are indeed major advancements recently on this line. A large variety of fluorescent proteins, self-labelling tags and bridging molecules has allowed the visualization of almost any molecular entity of the Central Dogma in living embryos by fluorescent imaging spanning a broad optical spectrum – from blue to infra-red (Tsien, 1998; Bindels et al., 2017; Shcherbakova et al., 2015; Los et al., 2008; Kolberg et al., 2013; Rodriguez et al., 2007; Tanenbaum et al., 2014). Second, new microscopes such as light-sheet microscopy and multiphoton microscopy, which significantly decrease photo-toxicity during imaging, have created unprecedented possibilities for long-term live imaging of embryonic development (Benninger et al., 2008; Wan et al., 2019). Third, advanced computational tools are needed and have been developed to extract biological relevant information from large imaging datasets (Amat et al., 2015). However, with of all these advancements, the first challenge we need to overcome for realizing all the promises of studying development in realtime, is still to obtain the embryo that carry the specific fluorescent reporters. It is actually quite challenging to obtain these reporter embryos in an efficient and scalable manner, especially for mammalian species such as mouse. This minireview will focus on the recent developments in the gene editing technology, including some of our own experiences, in efficiently generating knock-in reporter mouse models for live imaging research. This is not meant to be an exhaustive review of literature in knock-in technology, instead I will use a few approaches that have been relatively broadly implemented to illustrate the principles behind the new knock-in technologies to generate reporters. I regret not being able to discuss numerous interesting technology developments in this field.

Scientists have been generating reporter mice for a long time, mostly using two classical technologies. The first one is random transgenesis. In this case, protein dynamics were investigated through the use of fluorescent proteins driven by partial regulatory sequences such as promoter or enhancer regions of a gene-of-interest (Ikawa et al., 1998). These reporter constructs are integrated randomly into the mouse genome through pronuclear microinjection- a relatively fast process more amenable for large scale production (Ittner and Gotz, 2007). This approach has been instrumental in revealing gene expression patterns in live embryos. However, random transgenesis has intrinsic drawbacks. For example, random transgenic reporters frequently fail to recapitulate gene expression patterns due to the lack of full regulatory sequences. Also, the effects of local chromatin environment of the particular insertion site contribute to the expression variability between lines carrying the same transgene (Palmiter et al., 1983; Laboulaye et al., 2018; Weis et al., 1992). Moreover, recent efforts of mapping the insertion locus of a large number of established transgenic mouse lines has identified numerous accompanying complex genomic changes in addition to the transgene insertion. Such changes include large insertions or deletions at the insertion site, which could lead to confounding phenotypes unrelated to the particular transgene (Goodwin et al., 2019). Knock-in, defined as the precise insertion of an exogenous sequence into an endogenous genomic locus, could largely resolve these drawbacks and achieve faithful tagging of endogenous genes. Traditionally, knock-in mouse embryonic stem(ES) cells are first generated through homologous recombination mediated gene targeting, which typically has a low success frequency. Then these knock-in ES cells are aggregated with or injected into wildtype mouse preimplantation embryos to establish chimeric founders. Only when the knock-in ES cells contributed to the germline of the chimeric founders, knock-in mouse lines can be established. Therefore, it typically requires the screening of hundreds of ES cell clones, which is followed by time consuming chimera generation and breeding (Thomas and Capecchi, 1987; Capecchi, 2005). It is apparent that neither of these technologies would be sufficient for the precision and scalability of reporter generation if we are going to realize the promising of live imaging in developmental biology.

New genome editing technologies, most notably the CRISPR/Cas9 system, have completely changed the landscape in gene editing (Hsu et al., 2014). Particularly, it allowed targeted gene editing directly through micro-injecting into early embryos by targeted introduction of double strand breaks at desired genomic locus, which bypasses the typically tedious and time-consuming cell engineering steps (Yang et al., 2013). CRISPR/Cas9 zygote injection has been proved to be highly efficient in generating knock-out alleles through non-homologous end join(NHEJ) pathway, as well as generating point mutation and small tag insertions through homology directed repair(HDR) (Yang et al., 2013). These approaches have brought new hope for rapidly generating reporter embryos. However, although with initial promising reports, the efficiency of HDR mediated large fragment knock-in, which is required for inserting most fluorescent tags, through mouse zygote injection tends to be variable and not robust (Yang et al., 2013; Cohen, 2016). A few recently developed technologies have improved the efficiency of reporter mice generation.

The first was a technology called *E*fficient *a*dditions with *s*sDNA *i*nserts-CRISPR (Easi-CRISPR) (Quadros et al., 2017). Instead of double-stranded plasmid DNA that were traditional used for gene targeting, Easi-CRISPR uses long single-stranded DNA as donor templates (Quadros et al., 2017; Miura et al., 2018). By microinjection of ribonucleoprotein complexes of Cas9 protein bound with a complexed crRNA and tracrRNA guide alongside a long ssDNA template encoding the desired knock-in sequence flanked by 60 to 105 base homology arms, Easi-CRISPR was used to accomplish knock-in of fragments varying from 800 base pairs to 1.4 kilobase pairs with efficiencies ranging from 25-67% (Quadros et al., 2017). However, at present, long single-stranded DNA template synthesis remains a primary limitation of Easi-CRISPR, with insertion of fragments greater than 1-2 kilobases remaining a challenge. Furthermore, it has been observed that the secondary structure that may be formed within a long single-stranded DNA molecule could lead to the skipping of donor sequences during repair (Codner et al., 2018). This poses another challenge for precise knock-in using Easi-CRISPR.

A crucial technological advancement – two-cell homologous recombination  (2C-HR)-CRISPR – was provided by our group based on insights from the understanding of mouse early embryonic development (Gu et al., 2018). It is well-established that HDR is predominantly active in the late S-G2 phases of the cell cycle (Hustedt and Durocher, 2016). Indeed, there had been numerous reports that timing genome editing to coincide with this period *in vitro* could lead to significantly greater frequency of editing events mediated by HDR (Yang et al., 2016; Lin et al., 2014). We reasoned that the two-cell stage mouse embryo, which have an exceptionally long G2 phase (10-12 hours), might permit higher knock-in efficiency. Furthermore, the accessible chromatin state during zygotic genome activation, which occurs during the elongated G2 phase of two-cell embryos, could also contribute to efficient knock-in (Svoboda, 2018). Indeed, in a side-by-side experiment, delivery of the editing components to a two-cell stage embryo resulted in a significant enhancement of knock-in efficiency compared to zygotes, increasing the efficiency from 1-5% to 30-35% at the two loci tested. Among many gene locus, 2C-HR-CRISPR allowed for the knock-in of reporter alleles in up to 60% of founder mice – simply by changing the timing of delivery. Importantly, 2C-HR-CRISPR was shown to be highly efficient in spite of knock-in size and was used to introduce fragments as large as 7.5 kilobases. To further improve 2C-HR-CRISPR, we modified the system once further. There are speculations that HDR is hindered by the search for homologous sequences following the introduction of a DSB (Ma et al., 2017). Therefore, recruiting the donor template to the DSB-site might increase the efficiency of HDR. One efficient method to recruit DNA template to a Cas9 induced DSB is through the biotin-avidin interaction. Therefore, we constructed a Cas9-monomeric streptavidin(mSA) fusion gene. By co-injecting Cas9-mSA mRNA, sgRNA and a biotinylated PCR product as repair template, the repair template would bind to the Cas9-mSA protein and be recruited to the proximity of the double strand break and improve the knock-in efficiency. With this modification, the 2C-HR-CRISPR system produced knock-in efficiencies of up to 95% in some genes at the founder mouse stage (e.g., *Gata6-Halo* reporter gene) and high germline transmission – with some targets reaching 100%. Using 2C-HR-CRISPR, we have generated 35 knock-in reporters for critical developmental regulators, and moving forward to understand the developmental dynamics of mouse embryonic development. Some of these mouse models have been used to dissect the cell migration process in mouse gastrulation and the mechanical controls of mouse early limb development (McDole et al., 2018; Zhu et al., 2020).

While the techniques described above can reliably mediate large-fragment knock-in, in each case, their successful implementation all require micro-injection gene editing reagents into mouse early embryos. The technical challenges of micro-injection pose significant hurdle for their broad application. Recently, multiple groups have developed injection-free knock-in methods by delivering gene editing reagents using adeno-associated viral vectors into mouse zygotes (Chen et al., 2019; Yoon et al., 2018, Mizuno et al., 2018). A common theme of these techniques is the delivery of donor templates using AAV vectors, while the Cas9 and sgRNA are delivered either as AAV vectors or as Cas9-sgRNA complex (RNPs). These technologies have been shown to generate knock-in reporters with high efficiency (50%), therefore, greatly simplified the process of generating knock-in reporter mice. However, size of the knock-in cassette is limited by the packaging capability of AAV vector(4.9kb).

Other HDR independent technologies such as the micro-homology mediated end joining (MMEJ) mediated CRISPR/Cas9-based precise integration into the targeted chromosome (CRIS-PITCh) and homology mediated end joining (HMEJ) based methods has also been developed to generate knock-in reporters in a wide range of organisms (Nakade et al., 2014; Yao et al., 2017). These technologies as well hold great potential in generating knock-in reporter mice, although they are right now less used in mouse genetics, probably because the HDR based methods, that typically enable more sequence precision, are sufficiently efficient for mouse generation.

With these exciting technology developments, we are already seeing a number of reported studies dissecting mouse embryonic development using live imaging. With more and more knock-in reporters generated and characterized, we will enter a bright future of mammalian developmental biology.
