# ERRATUM

**DOI:** 10.1590/1984-3143-AR2020-0078

**Published:** 2020-09-17

In the article entitle “Light up the embryos: knock-in reporter generation for mouse developmental biology”, DOI number: https://doi.org/10.1590/1984-3143-AR2020-0055, published in journal Animal Reproduction, 2020, volume 17, number 3, page 1:

Where it reads:

“Thematic Section: 36th Annual Meeting of the Association of Embryo Technology in Europe (AETE)”

It should read:

“Thematic Section: 34th Annual Meeting of the Brazilian Embryo Technology Society (SBTE)”

